# An advanced structural characterization of templated meso-macroporous carbon monoliths by small- and wide-angle scattering techniques

**DOI:** 10.3762/bjnano.11.23

**Published:** 2020-02-10

**Authors:** Felix M Badaczewski, Marc O Loeh, Torben Pfaff, Dirk Wallacher, Daniel Clemens, Bernd M Smarsly

**Affiliations:** 1 Institute of Physical Chemistry, Justus Liebig University, Heinrich-Buff-Ring 17, 35492 Giessen, Germany; 2 Schunk Carbon Technology GmbH, Rodheimer Straße 59, 35452 Heuchelheim, Germany; 3 Helmholtz-Zentrum Berlin für Materialien und Energie, Hahn-Meitner-Platz 1, 14109 Berlin, Germany; 4 Center for Materials Research (LaMa), Justus-Liebig-University, Heinrich-Buff-Ring 16, 35392 Giessen, Germany

**Keywords:** adsorption, carbon materials, mesoporosity, microporosity, microstructure, pore structure, small-angle neutron scattering (SANS), wide-angle X-ray scattering (WAXS)

## Abstract

This study is dedicated to link the nanoscale pore space of carbon materials, prepared by hard-templating of meso-macroporous SiO_2_ monoliths, to the corresponding nanoscale polyaromatic microstructure using two different carbon precursors wthat generally exhibit markedly different carbonization properties, i.e., a graphitizable pitch and a non-graphitizable resin. The micro- and mesoporosity of these monolithic carbon materials was studied by the sorption behavior of a relatively large organic molecule (*p*-xylene) in comparison to typical gas adsorbates (Ar). In addition, to obtain a detailed view on the nanopore space small-angle neutron scattering (SANS) combined with in situ physisorption was applied, using deuterated *p*-xylene (DPX) as a contrast-matching agent in the neutron scattering process. The impact of the carbon precursor on the structural order on an atomic scale in terms of size and disorder of the carbon microstructure, on the nanopore structure, and on the template process is analyzed by special evaluation approaches for SANS and wide-angle X-ray scattering (WAXS). The WAXS analysis shows that the pitch-based monolithic material exhibits a more ordered microstructure consisting of larger graphene stacks and similar graphene layer sizes compared to the monolithic resin. Another major finding is the discrepancy in the accessible micro/mesoporosity between Ar and deuterated *p*-xylene that found for the two different carbon precursors, pitch and resin, which can be regarded as representative carbon precursors in general. These differences essentially indicate that physisorption using probe gases such as Ar or N_2_ can provide misleading parameters if to be used to appraise the accessibility of the nanoscale pore space.

## Introduction and Motivation

Porous sp^2^-hybridized carbon materials are frequently used in various applications such as supercapacitors or batteries for the storage of electric energy, as filters for the purification of air or water, and in adsorption processes [1–8]. The turbostratic microstructure of these “non-graphitic” carbon materials combined with variable conductivity and a significant nanoscale disordered porosity are typical features of this kind of carbon materials. Templating strategies are meanwhile well established to endow carbon materials with defined porosity on the nanometer scale, in particular to enhance the surface area and to control the pore size [9–12]. This study is dedicated to a quantitative determination of the porosity, e.g., pore size, pore volume and pore shape of carbon materials prepared by hard-templating of meso-macroporous SiO_2_ monoliths, and to a linkage of these parameters to the corresponding nanoscale polyaromatic microstructure. Usually, temperature treatment at elevated temperatures needs to be applied to carbonize molecular organic substances, but the commonly applied temperatures of 800–3000 °C can markedly affect the nanoscale porosity [13–21]. Hence, a combined in-depth elucidation of meso/microporosity and the graphene-based structure upon heat treatment is pursued in this study to obtain a profound understanding of the relationship between the changes in the nanopore space and the building blocks surrounding the nanopores.

In essence, the structure of “non-graphitic carbon materials” consists of hexagonal graphene layers that are stacked in a parallel way but do not possess 3D long-range crystallographic order. This structure is referred to as “turbostratic” arrangement. These graphene stacks represent the basic structural unit (BSU) of non-graphitic carbon materials and exhibit a low packing density [21–24]. The microporosity (pores below 2 nm) results from imperfections in the packing of the graphene stacks and should thus be dependent on the degree of disorder in the material. That is, with increasing disorder of a material a higher micropore volume is expected. Because with higher carbonization temperature the graphene sheets as well as their stacking become more ordered, the meso/microporosity is expected to depend on the heat treatment temperature.

The degree of disorder in the graphene stacks can be determined by evaluating wide-angle X-ray (WAXS) and neutron (WANS) scattering data of turbostratic carbon materials. Several current evaluation methods for WAXS and WANS are based on the fitting of entire WAXS/WANS data sets using suitable theoretical scattering functions under variation of structural parameters [25–29]. Here we apply Ruland’s and Smarsly’s model allowing for the in-depth evaluation of such scattering data, which describe the carbon microstructure on an angstrom scale [30–32].

In addition, a dependency of the porosity on the precursor materials is expected as some materials are better graphitizable than others. For instance, resin-based carbon materials are not graphitizable, while pitch-based carbon materials develop a comparably high structural order upon heat treatment and can be converted into graphite.

The main approaches to influence the carbon structure are the choice of the carbon precursor and the applied heat treatment temperature for carbonization or graphitization. These two factors have the highest impact on the resulting sp^2^-hybridized microstructure. Since the porosity mainly consists of microporosity additional approaches are necessary to further introduce mesoporosity or macroporosity and to tune the pore system. Chemical and physical activation, using reactive agents such as bases (KOH) or gases (CO_2_), are only capable to enhance the microporosity by etching the carbon skeleton [33–34]. To create meso- or macropores into the carbon system templating approaches have become a routine strategy. One prominent attempt is hard-templating based on silica monoliths with a bimodal pore size distribution (meso- and macropores) and a hierarchical pore network [35–38]. The SiO_2_ solid is impregnated with a liquid or dissolved carbon precursor, and thus the carbonization of the precursors takes place within the pores. Template removal is typically performed with concentrated aqueous solutions of NaOH or HF. The hierarchical pore system and the bimodal distribution of pore sizes provide a high surface area and a high permeability. Due to big macropores the material can achieve high flow rates for separation processes, and the mesopores within the walls of the macropores lead to a high surface area, which is beneficial for adsorption processes [39–40]. Carbon replicas based on silica monoliths are potential candidates for lithium or lithium–sulfur battery systems, in which the carbon acts as a conductive matrix [41–43]. Other important features for this kind of applications are the connectivity and the accessibility of the pore network. The connection between large and small pores can lead to unfavorable bottlenecks. These narrow bottlenecks can act as a barrier for larger molecules, which block the pore access and lower the pore connectivity. Therefore, we want to investigate the adsorption behavior of monolithic carbon materials for larger molecules in comparison to typical gas adsorbates such as nitrogen, argon, krypton or carbon monoxide. We chose *p*-xylene as an adsorbate for vapour sorption to address the sorption at room temperature.

To obtain a detailed view of the nanopore space, which exhibits micro-, meso- and macropores, but with an upper limit of ca. 100 nm, small-angle neutron scattering (SANS) combined with in situ physisorption were the methods of choice. Deuterated *p*-xylene (DPX) acts as a contrast-matching agent in the neutron scattering process. In a perfect case, that is if DPX filled all pores, the scattering contrast would be zero and the SANS intensity would vanish. Hence, performing SANS coupled with an in situ physisorption experiment allows for investigating the pore-filling process. In this study we compare the pore network of different carbon monoliths in an empty state and a filled state. The SANS data were analyzed quantitatively in terms of pore size, porosity, wall thickness and angularity of the pores. In addition, the approach of the chord-length distribution (CLD) is used [44–48].

Another question we want to address is the impact of the carbon precursor on the structural order on an atomic scale in terms of the size and the disorder of the carbon microstructure, on the nanopore structure, and on the template process. A graphitizable pitch and a non-graphitizable resin, which carbonization turns into a glassy carbon, is used for comparison. Glassy carbon materials are derived from thermally processed phenolic formaldehyde resins, which often exhibit a closed porosity (voids). Resins are important compounds in the production of many carbon materials, e.g., as binder matrix for carbon fiber-reinforced carbon materials (CFRC), a light-weight material with excellent mechanical properties even at high temperatures. Upon heat treatment, the PF resin is gradually transformed into a non-graphitizing glassy carbon [49–50] consisting of highly cross-linked graphene stacks, which once were thought to be ribbon-like, but are now acknowledged as being of made of highly curved graphene sheets with a high content of fullerene-like structures [51–54]. Key properties of glassy carbon materials, such as thermal conductivity, chemical resistance, hardness, density, and coefficient of thermal expansion are closely related to the carbon microstructure and the porosity. Resin-based carbon materials are known to possess a substantial content of inaccessible voids on the nanometer scale, in addition to accessible pores [53–55]. Thus, in situ SANS experiments can help to differentiate between the inaccessible and accessible voids in such template carbon materials. Here, we compare templated carbon materials obtained at 800 and 3000 °C, for which in situ SANS experiments were performed comparing the evacuated samples with the filled state. A temperature of 3000 °C was chosen, because at this treatment temperature graphitized carbon forms, which possesses advantageous properties such as high electronic conductivity. The porosity of such materials having undergone a treatment at lower temperatures was also addressed by us in a recent study [56].

The microstructure of both carbon materials is investigated by an advanced wide-angle X-ray scattering approach. Since standard scattering evaluation methods such as single-peak analysis or Rietveld refinement suffer from the turbostratic arrangement of the carbon stacks and do not achieve meaningful results, a novel approach similar to Rietveld refinement is used [28]. The algorithm of the approach is capable to fit the entire wide-angle scattering curve of turbostratic carbon materials. Physically meaningful parameter such as the graphene layer size *L*_a_, the stack size *L*_c_, the carbon bond length *l*_cc_, the layer distance *a*_3_, and the disorder parameter describing the disorder within the layers (σ_1_) and between the layers (σ_3_) are directly quantified and can be compared.

All in all, in this study advanced structural characterization methods are used to quantify the microstructure and pore structure of carbon monoliths based on different kinds of carbon precursors, namely a graphitizable pitch and a non-graphitizable resin. This coherent analysis helps to understand the impact of the precursor on the templating process and the final porosity, in relation to the formation of sp^2^-hybridized microstructures.

## Results and Discussion

### Characterization of the pore system

This study is focused on four different hard-templated carbon monoliths based on pitch or resin as carbon precursor. In both cases, the hard-templated carbon was exposed to two substantially different temperatures, namely 800 and 3000 °C. This means that two porous carbon materials were treated at 800 and two at 3000 °C. In short, in the hard-templating process the pristine silica monoliths were infiltrated by a liquid coal-tar pitch (“Pitch”) or a liquid resole (“Resin”). The carbonization was conducted under nitrogen atmosphere and at a maximum heat treatment temperature of 800 °C. The SiO_2_ template was etched by exposure to hydrofluoric acid, and the graphitization took place in an Acheson furnace at 3000 °C. The bimodal meso-macropore structure of these four carbon materials as well as of the meso-macroporous SiO_2_ monolith template was investigated by scanning electron microscopy (SEM), mercury intrusion porosimetry (MIP) and physisorption.

**Figure 1:**
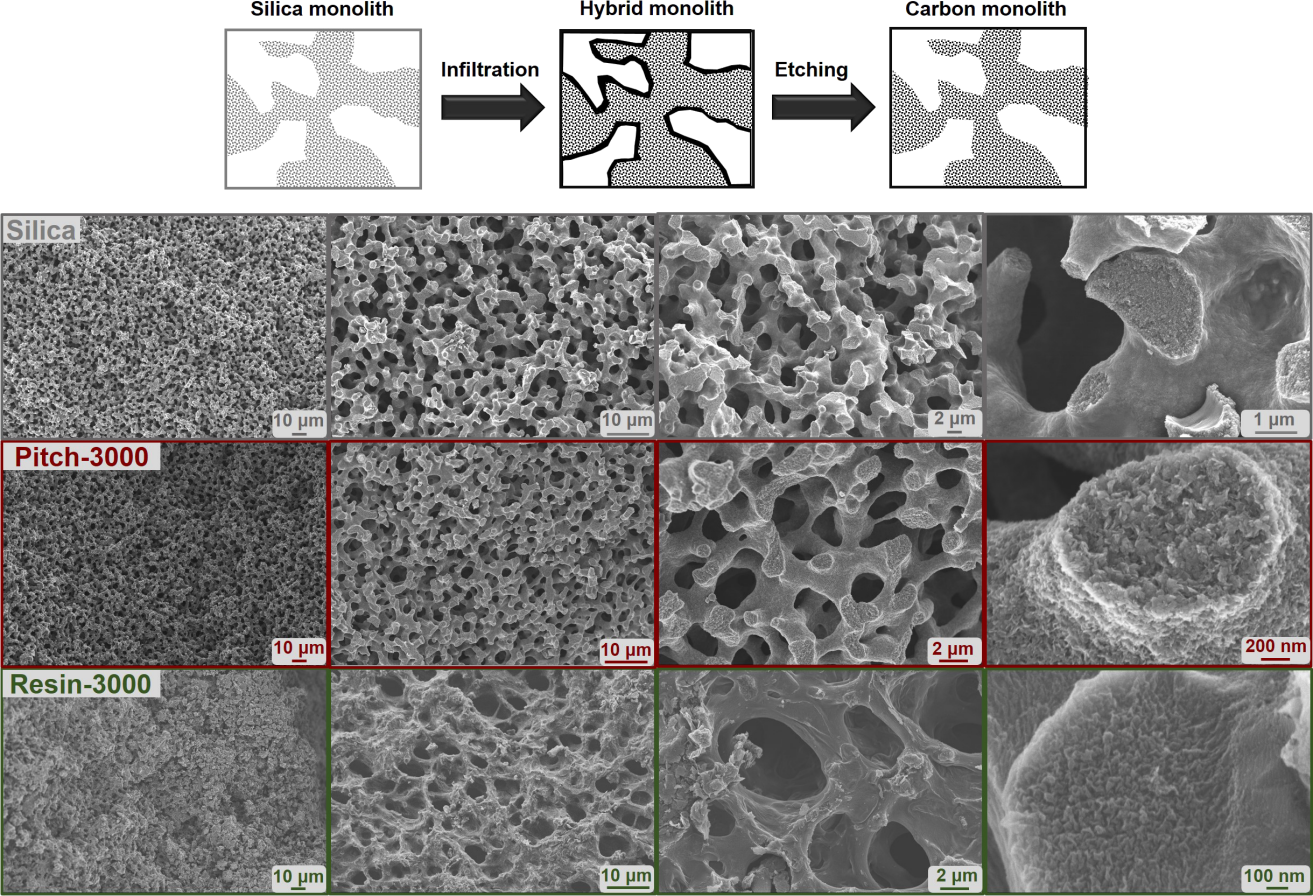
Scheme of the template process (top). SEM pictures of the silica template (grey), pitch-based (red) and resin-based (green) monoliths carbonized at 3000 °C.

Figure 1 shows SEM images of the monolithic silica (grey) as well as the pitch (red) and the resin (green) graphitized at 3000 °C. The sponge-like monolithic structure is retained even at high temperature. The resin-derived carbon monolith displays thicker macropore walls and a less homogenous macropore space, while the macroporosity of the pitch-based carbon monolith looks almost identical to that of the original SiO_2_ monolith. Since the macropore dimensions are almost identical in the SiO_2_ template and in the resulting carbon, the templating process is mainly dominated by a covering of the macropore walls by pitch molecules rather than a complete filling of the macropore space. A 1:1 copy of the pristine SiO_2_ macropore space is obtained (Figure 1, top). In case of the mesopores the filling is based on a substantial capillary pressure, capturing the nonpolar liquid precursor within the mesopores, which are then replicated. The corresponding SEM images after heat treatment at 800 °C are given in Supporting Information File 1.

**Figure 2:**
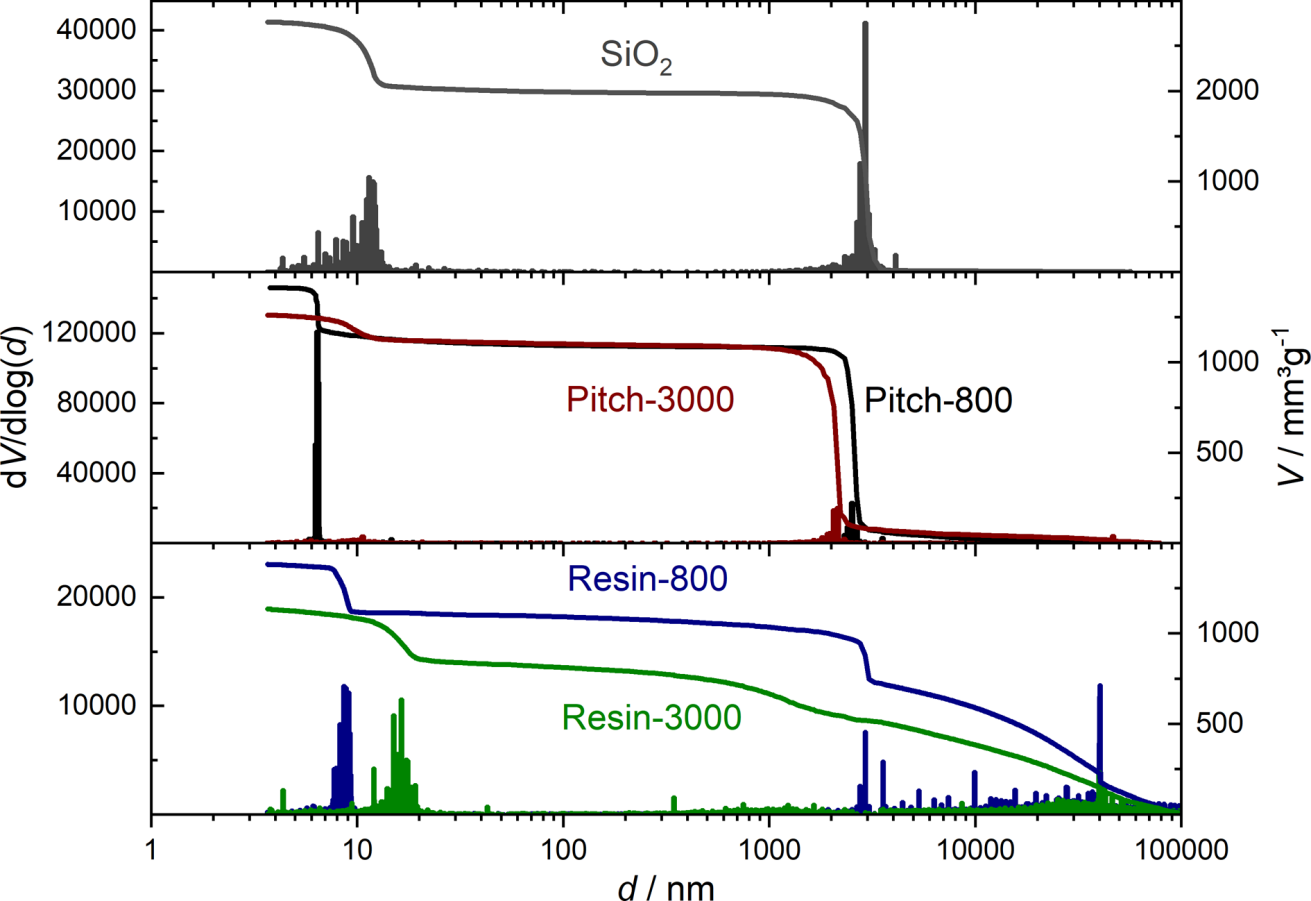
Mercury intrusion porosimetry (MIP) data of the pristine meso-macroporous SiO_2_ monolith (top) and the templated carbon materials, treated at 800 and 3000 °C.

The initial bimodal pore size distribution of the SiO_2_ features mesopores of 13 nm and macropores of 3 μm in diameter (Figure 2). In the case of the pitch-based sample carbonized at 800 °C (black), the mesopore size of 7 nm is smaller than that of the initial template, corresponding to the average thickness of the walls separating the mesopores in the original SiO_2_ monolith. A slight shrinkage in macropore size from 2.5 μm to 2 μm is observable for the graphitized pitch sample (Figure 2, Pitch-3000) compared to the Pitch-800 monolith.

The macropore size distribution of the resin-based monoliths is very broad compared to that of the pitch-based carbon materials, probably due to experimental issues: The very rigid and stiff resin-based monoliths were crushed by a ball mill in order to fit into the sample holder for MIP measurements. Consequently, interparticular voids appear in the MIP analysis, in addition to the template macroporosity. Nevertheless, the step in the cumulative pore volume at 2 μm is attributable to the dominating macropore size, which corresponds to the expected dimension. Mesopores of 9 nm in diameter are observed in the resin-based carbon treated at 800 °C, shifting to higher values during heat treatment, contrary to the pitch-based carbon material. The increase in the mesopore diameter observed at 3000 °C (Resin-3000) is attributable to the merging and growth of graphene stacks that undergo sintering, thus removing smaller mesopores and leaving behind larger mesopores. Also, the mesopore size distributions of the carbon monoliths at 800 °C are very narrow, which indicates that the SiO_2_ walls separating the mesopores exhibit a quite uniform thickness.

**Figure 3:**
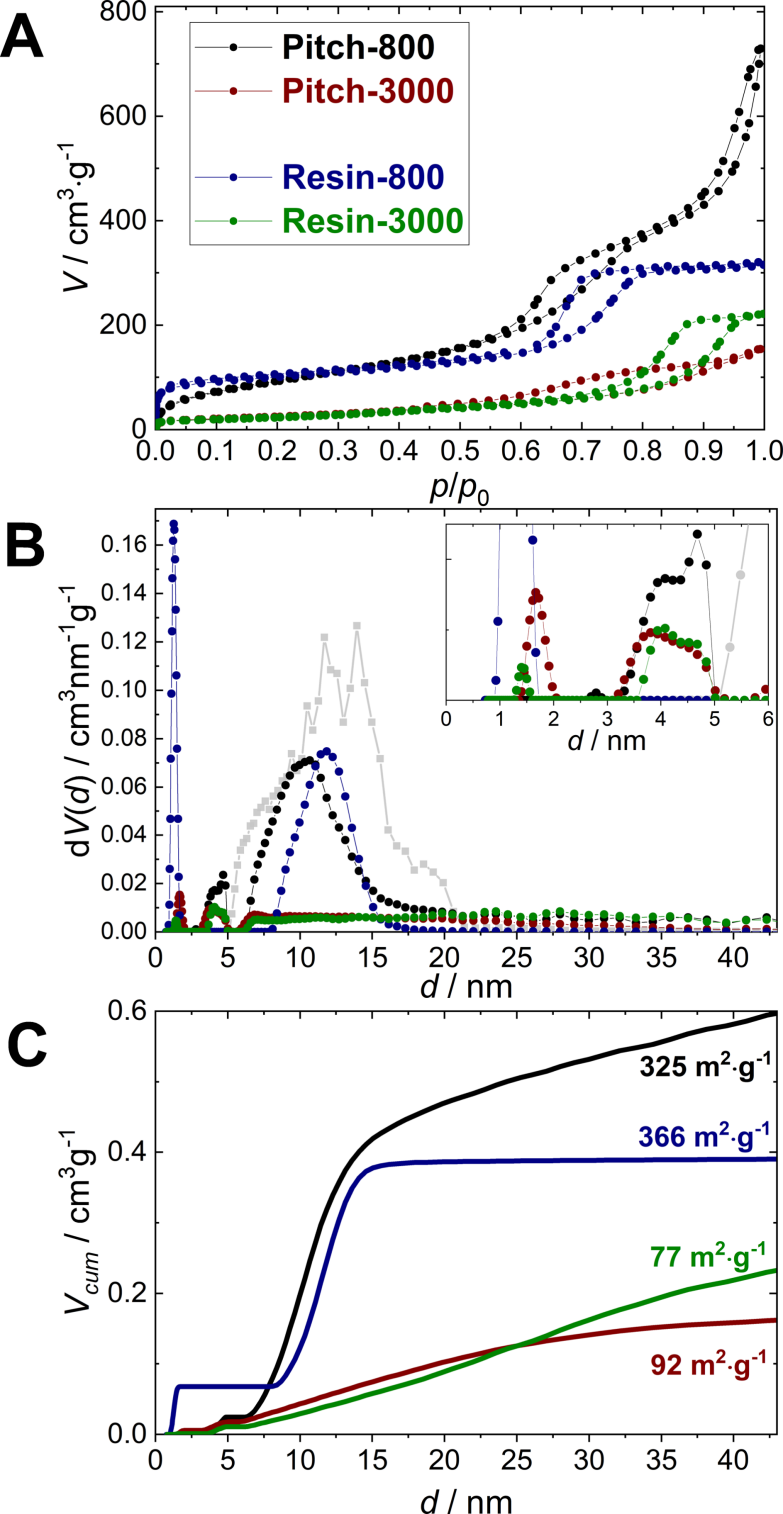
Argon isotherms at 87 K (A), pore-size distributions of the silica template (grey) and the templated carbon materials (B) and cumulative pore volumes (C).

Figure 3 shows Ar sorption isotherms (87 K) and the resulting pore size distributions and cumulative pore volumes. All samples show type-IVa isotherms indicating a defined mesopore space [57].

The two replicas carbonized at 800 °C show a narrow mesopore size distribution at around 13 nm (Pitch-800) and 9 nm (Resin-800), and BET surface areas larger than 300 m^2^·g^−1^ in both cases. Small mesopores (4 nm) and also micropores are detectable for all samples, while Resin-800 shows pronounced microporosity, which is typical of resin-derived carbon materials. The heat treatment at 3000 °C decreases the mesopore volume and surface area significantly for both precursors and the mesopore size distribution broadens substantially. The BET surface area values drop to about 90 m^2^·g^−1^ for both carbon precursors at 3000 °C. The micropore volume is slightly larger for Pitch-3000 compared to Resin-3000. One reason for the difference can be closed voids in the resin structure that are inaccessible for Ar atoms.

**Figure 4:**
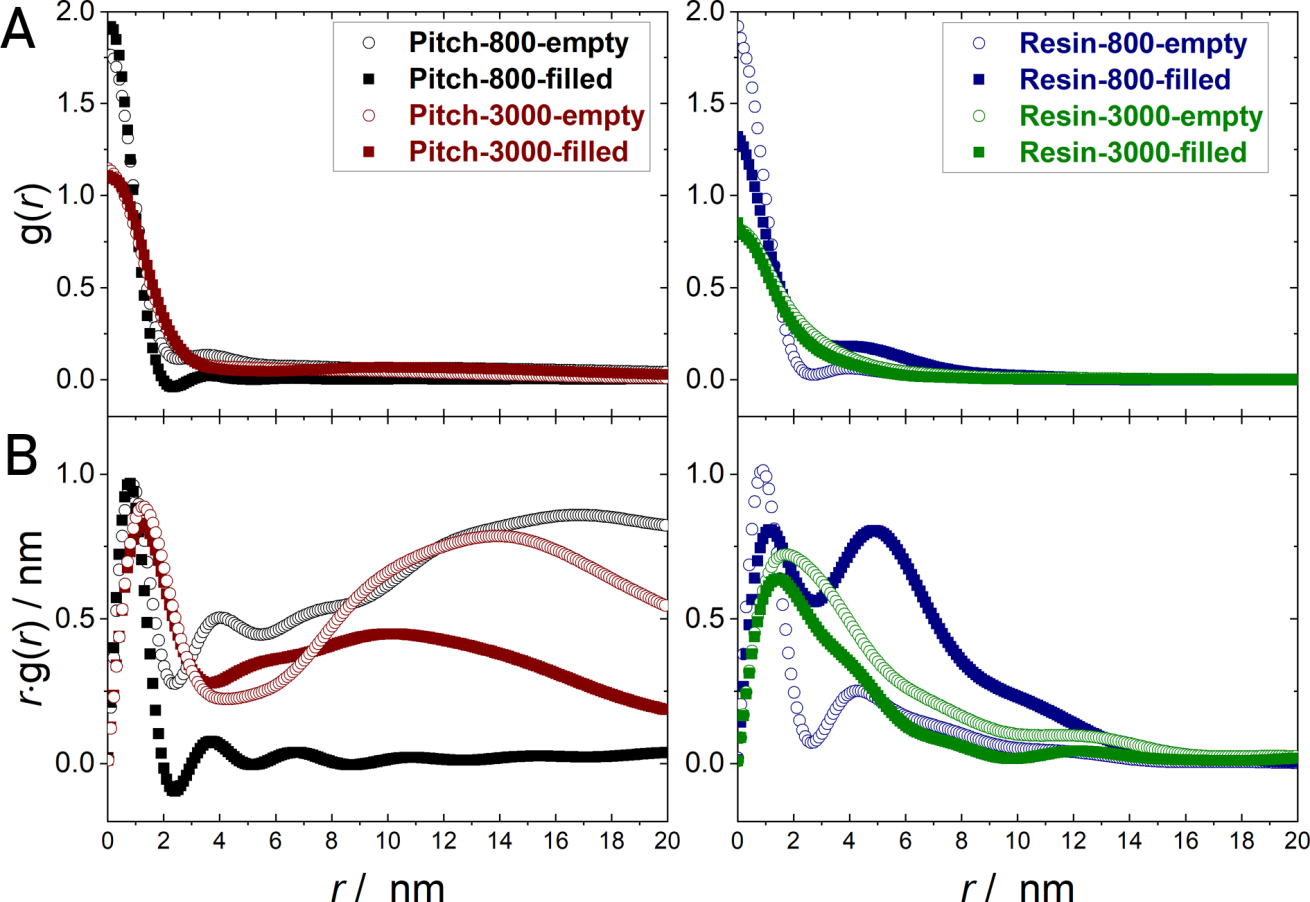
SANS raw data (A) and CLD analysis (B) for the four resin- and pitch-based carbon materials, treated at 800 and 3000 °C. For all samples, SANS analysis was performed on evacuated samples (hollow symbols), as well as under a maximum load of deuterated *p*-xylene (filled symbols). All samples exhibit a Porod-asymptote (*s*^−4^) at large *s* (modulus of the scattering vector), proving an almost ideal two-phase system (pore–carbon) with sharp interfacial boundaries on the nanometer scale.

Since carbon materials can possess a significant degree of inaccessible voids on the nanoscale, small-angle scattering is a suitable method to determine the overall nanoscale porosity and hence to determine the fraction of inaccessible voids. Figure 4 displays the acquired SANS data of the monolithic carbon materials in an empty state and filled with a maximum load of DPX. The SANS intensity arises due to the contrast of the neutron scattering length density between the carbon matter and the pores and/or voids. DPX and carbon possess almost identical scattering length densities with respect to neutrons, thereby enabling the principle of “contrast matching”, i.e., micro- and mesopores filled with *p*-xylene no longer contribute to the SANS pattern, and the filling process can thereby be studied by comparing the SANS patterns of the filled material and the initial empty sample. The impact of the graphitization process on the pore sizes is directly observable by comparing the SANS curves in a qualitatively manner. The pitch-based monolith carbonized at 800 °C shows higher SANS intensities in the *s*-range corresponding to microporosity (0.7–0.9 nm^−1^) than the 3000 °C pitch sample. The same trend is observable for the monolithic resins. The resin carbonized at 800 °C shows the highest SANS intensity originating from micropores (at large *s*-values), which is in good agreement with the physisorption analysis. For the mesoporosity the opposite trend is noticeable, the absolute intensity in the corresponding SANS range increases with heat-treatment temperature, which also meets the physisorption analysis.

Interestingly, the SANS curves of the filled samples do not differ significantly from the curves of the empty samples. The absolute SANS intensity decreases due to the contrast matching adsorbed DPX and the carbon skeleton, but does not vanish as would be expected for a complete filling of the pores. The SANS intensity at very low (ca. 0.008 nm^−1^) and very high (ca. 0.1 nm^−1^) *s*-values does not change upon exposure to DPX, which means small micropores and larger mesopores and macropores are not filled. In contrast, a certain fraction of mesopores between ca. 5 nm and ca. 50 nm is occupied by DPX. These observations are different for the Resin-800 sample. Here, the SANS intensity increases markedly during vapor sorption. A high number of inaccessible voids and sealed porosity hinders the penetration of the pore network, and the adsorption of DPX on the external surface leads to a larger scattering contrast, which in turn increases the overall intensity.

The chord-length distributions *g*(*r*) (Figure 4B) were obtained by fitting the SANS data with a recently introduced parametrization approach [48]. The CLD evaluation allows for the characterization of disordered pore systems and is suitable for two-phase systems, i.e., carbon matter and pores in this case. It provides a distance distribution of two connected phase boundaries. The function *g*(*r*) consist of contributions from pores and matter and can therefore, in general, not be directly related to the pore space. The contributions of the matter phase dominate *g*(*r*) for highly porous materials, while *g*(*r*) correlates to the pore space in the case of low-porosity materials. In the latter case *g*(*r*) corresponds to a pore size contribution.

The representation *r*·*g*(*r*) is more appropriate to illustrate the dominating length scales, as the first moment of *g*(*r*) (Porod length, *l*_p_) is defined as


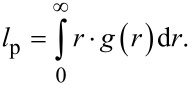


The *r*·*g*(*r*) curves of the pitch-based sample indicate the filling of mesopores of diameters above ca. 8 nm, i.e., the corresponding contribution decreases from the empty to the filled state. In the region *r* < 2 nm no significant decrease is observable, hence no corresponding pores are filled. For the sample Resin-800 the trend is different. Micropores and small mesopores are filled, but the SANS data themselves indicate that only a small fraction of the pores is accessible to DPX. One possible reason is a significant portion of isolated voids, not being accessible for DPX, which is well known for resin-based carbon. Since micropores are filled and mesopores are not, the relative contribution of mesopores to *g*(*r*) increases. Table 1 shows parameter directly derived from the SANS data measured in absolute scattering intensities. The parameters are defined in Equations S1–S10 of Supporting Information File 2.

**Table 1:** Summary of SANS analysis and SANS-derived porosity parameters for the different samples under study.

	Pitch-800-empty	Pitch-800-filled	Pitch-3000-empty	Pitch-3000-filled	Resin-800-empty	Resin-800-filled	Resin-3000-empty	Resin-3000-filled

porosity *P*	0.34	0.05	0.11	0.07	0.14	0.14	0.11	0.05
Porod length *l*_p_ / nm Equation S6	4	2	3	5	1	3	2	2
*l*_p,g(_*_r_*_)_ / nm Equation S5	4	1	3	4	1	2	2	2
angularity *g*(0)	1.8	1.9	1.1	1.1	1.9	1.3	0.8	0.9
average pore size *l*_pores_ / nm	7	2	4	6	1	3	2	2
average wall dimension *l*_matter_ / nm	1.3	4.2	4.4	12	0.6	1.7	4.7	9.3
average stack height *L*_c,WAXS_ / nm	1.2	—	n.a.	—	0.9	—	n.a.	—
Surface *S*/*V* / m^2^∙cm^−3^	305	92	85	29	649	228	81	42
polydispersity in the pore shape *k*_l_	2	4	2	4	4	1	2	3

The porosity *P* decreases due to the DPX adsorption except for Resin-800, where closed porosity, inaccessible voids and small micropores hinder the penetration of the pore network by DPX. The porosity of the sample Pitch-800 is higher than that of the other samples, which is in good agreement with the physisorption results. The pore size distribution determined by physisorption shows the existence of larger mesopores contributing to the porosity. The average chord length *l*_p_ (Porod length) was calculated by two approaches, namely from *g*(*r*) using the approach in Equation S5 (Supporting Information File 2) and from the SANS data themselves suing Equation S6 (Supporting Information File 2) [48,56]. The two values are in good agreement for all samples, which shows the validity of the two approaches for the elucidation of SANS data. The *g*(0) value is an indicator for the angularity of pores, where high *g*(0) values indicate sharp edges. When changing the heat-treatment temperature from 800 to 3000 °C the pore shape gets smoother, since the *g*(0) values decrease.

The SANS-derived pore sizes *l*_pores_ of all samples are below 10 nm and do not change significantly during the DPX vapor adsorption, because apparently a large fraction of the voids is inaccessible porosity. The overall average wall dimension *l*_matter_ increases due to the adsorption of DPX, proving that adsorption takes place. Interestingly, *l*_matter_ lies in the range of the graphene stack heights *L*_c_ analyzed by wide-angle X-ray scattering (WAXS, see next section), meaning the pores are separated only by one or two graphene stacks and the graphene layers face to the pores. The surface area *S*/*V* decreases during vapor sorption for all samples. Resin-800-empty shows the highest *S*/*V* values due to a high number of voids and micropores. This value is quite high in the filled state, because of inaccessible micro- and mesopores.

An increase in the polydispersity of the pore shape *k*_l_ upon DPX sorption, as seen for Pitch-800, indicates that the shape of the pores gets more inhomogeneous due to a not fully homogenous covered adsorption. This surprising finding is attributed to an inhomogeneous distribution of accessible and inaccessible voids. For Resin-800 the parameter *k*_l_ decreases, which indicates that the inaccessible pores or closed voids are quite homogenous in their shape.

**Figure 5:**
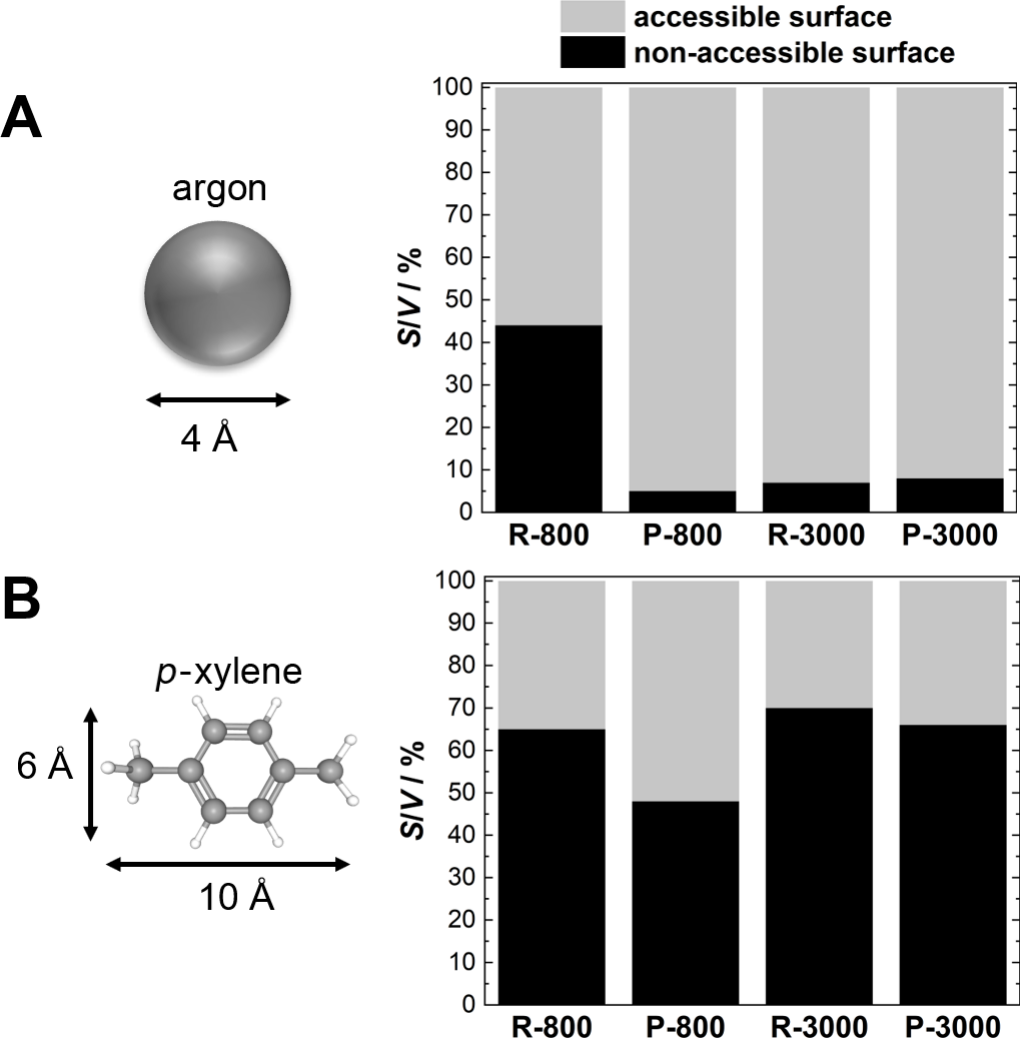
(A) Ratios of the surface areas are based on Ar physisorption and SANS analysis (empty samples) (*S*_Phys.,Ar_/*S*_SANS,empty_). (B) Ratios based only on SANS analysis of the empty and DPX-filled carbon samples (*S*_SANS,filled_/*S*_SANS,empty_).

To further investigate the surface area in terms of accessibility the results from the SANS and physisorption analysis are compared in a semi-quantitatively way. The obtained values of the argon BET surface (Figure 3C) and the overall SANS *S*/*V* surface (Table 1) give insights into the accessibility of the pore network upon adsorption. Figure 5A shows the proportion of the surface obtained by argon physisorption in comparison to the overall surface obtained by SANS of the empty samples. This comparison shows that for the Resin-800 sample the SANS-derived surface is much higher than the surface derived from Ar physisorption. For this sample more than 40% of the overall surface area is not accessible to Ar atoms. Figure 5B shows the surface values obtained from only the SANS analysis of the empty and DPX-filled samples. Here the non-accessibility (black) is much more pronounced. The available surface area for DPX adsorption is less than 50% for all carbon samples.

### Microstructural characterization

**Figure 6:**
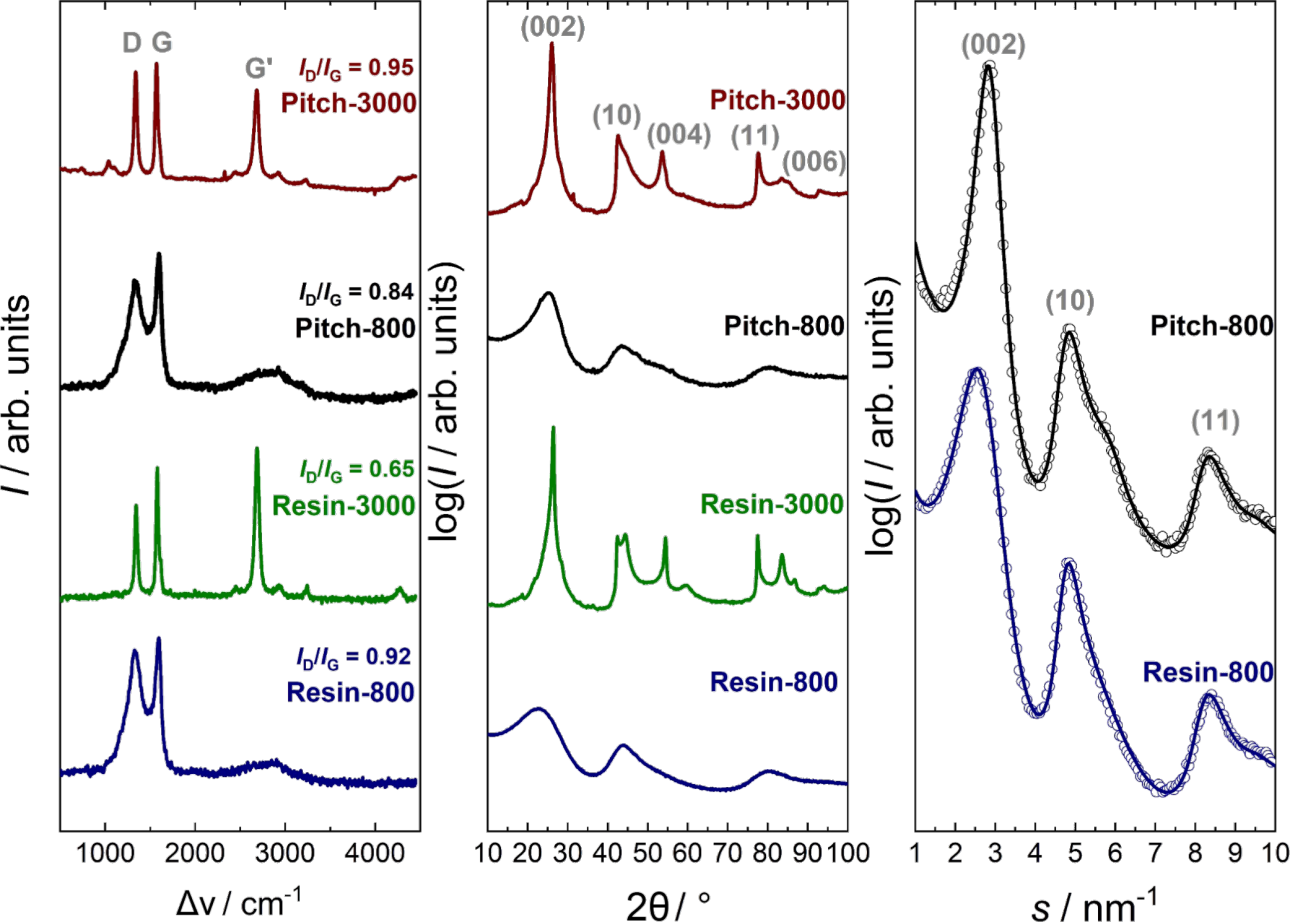
Raman data, WAXS data and fits (lines) of the WAXS data (open circles).

Figure 6 depicts the Raman raw data of the monolithic carbon materials. The disorder-induced D band arises from breathing vibrations of carbon rings and the G band resulting from carbon-chain vibrations prove the sp^2^-hybridized turbostratic microstructure. The G′ band is an overtone, where two phonons are involved. It scales with the number of layers in a graphene stack. A higher intensity indicates a growth of the stacks. The overlap of the D and G band vanishes at 3000 °C, which is a result of a higher structural order within the graphene layers.

The WAXS data (Figure 6) show the typical scattering maxima of turbostratic carbon materials. At 800 °C there are only three very broad reflections in the whole 2θ range from 10° to 100°. The (002) reflections arises due to the interlayer scattering between parallel stacked carbon layers. The (10) and (11) signals arise from the intralayer scattering within the graphene layers. Interestingly, the template resin- and pitch-based samples treated at 3000 °C show no general (*hkl*) reflections as expected for graphite, while the non-templated precursors are converted to graphite at this temperature. This absence of 3D order can be attributed to the confinement and the nanostructure, which hinder the rearrangement and growth of the stacks. We applied the algorithm by Ruland and Smarsly to fit the WAXS data in the whole scattering range and to obtain relevant microstructural parameters. However, the WAXS data of the 3000 °C samples cannot be analyzed by such approaches, because the carbon is “graphitic”, i.e., the structure is between that of turbostratic carbon and that of graphite. Additional reflexes in the range of the (10) maxima shine through, which are due to impurities, but are negligible in the analysis. Since the applied model is only applicable for turbostratic carbon materials, only the 800 °C samples were fitted. The fit of the two scattering curves is in very good agreement with the experimental data, providing reasonable values for different structural parameters (Figure 6). The obtained parameters thus allow for a comparison of the two carbon precursors with respect to the graphene stacks (Table 2).

**Table 2:** WAXS parameter for the pitch- and resin-based carbon materials. The parameter based on the corresponding non-templated bulk materials are shown in brackets.

	Resin-800	Pitch-800	Resin-3000	Pitch-3000

*L*_a_ / nm	1.9 (1.9)	1.6 (1.9)	19 (12)	10 (38)
σ_1_	0.14 (0.14)	0.12 (0.14)	—	—
*l*_cc_ / nm	0.141 (0.141)	0.142 (0.141)	—	—
*L*_c_ / nm	0.9 (0.7)	1.2 (1.2)	14 (2.3)	8 (30)
σ_3_ / nm	0.071 (0.047)	0.038 (0.027)	—	—
*a*_3_ / nm	0.379 (0.359)	0.350 (0.344)	0.338 (0.340)	0.342 (0.336)
*N*	2 (2)	4 (4)	41 (7)	29 (89)

**Figure 7:**
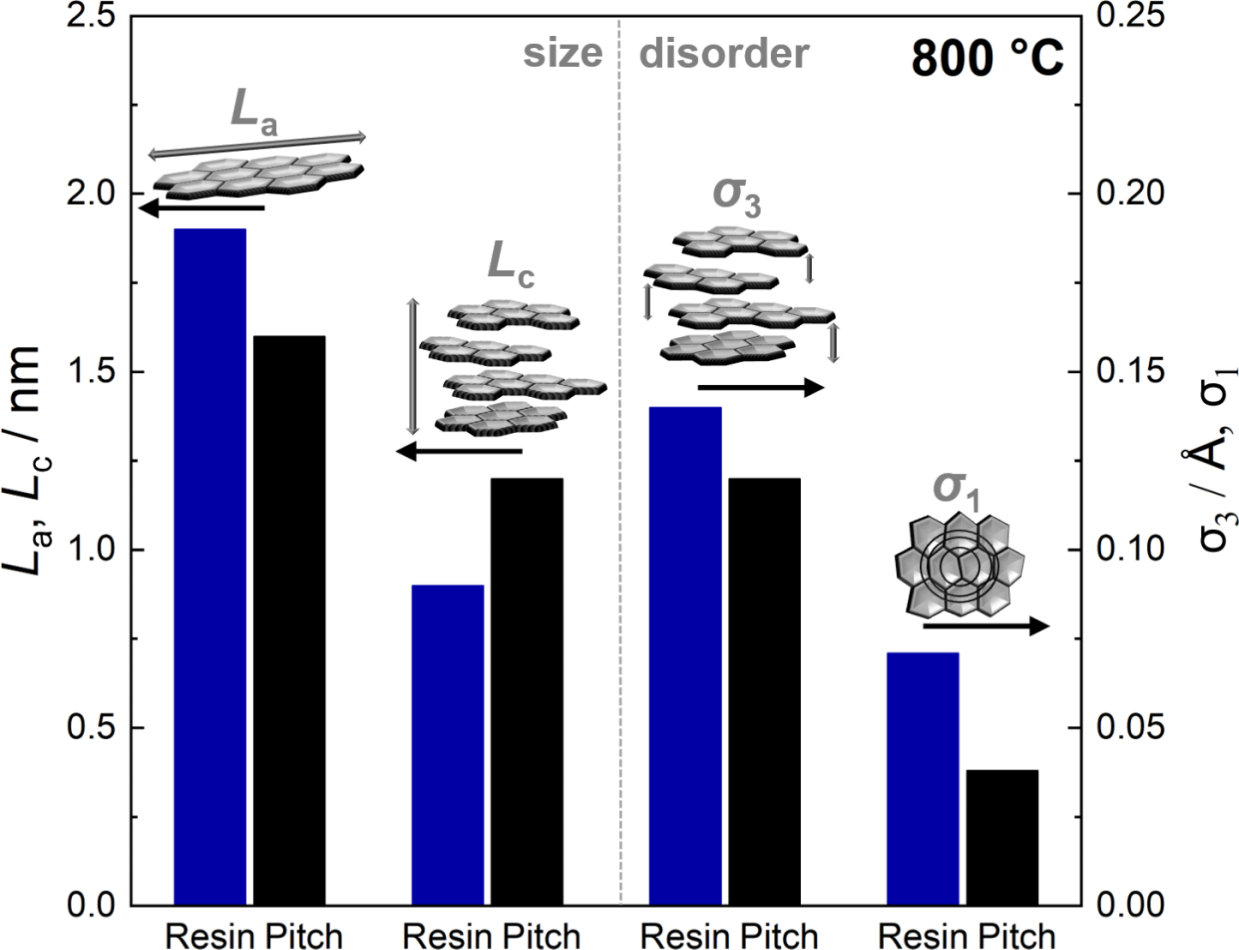
Microstructural parameters describing the size and disorder of graphene-like structures. The values are obtained from templated resin (blue) and pitch (black) carbonized at 800 °C.

Table 2 and Figure 7 show that the graphene layers of the resin (*L*_a_ = 1.9 nm) microstructure are slightly larger those of the pitch (*L*_a_ = 1.6 nm). Larger differences occur in the formation of the graphene stacks. By contrast, the stack height *L*_c_ is larger (1.2 nm) for the pitch samples compared to the resin samples (0.9 nm). The monolithic resin exhibits only two layers per stack on average, which are separated by a rather large distance of 0.379 nm. For the pitch-based monolith, the numbers of layers per stack of the pitch is twice as high and the layers in the stacks are packed denser (*a*_3_ = 0.350 nm). Figure 7 shows that the disorder within the graphene sheets (σ_1_) and within the stacks (σ_3_) of the resin monolith (σ_1_ = 0.14, σ_3_ = 0.071 nm) is significantly higher than that of the pitch monolith (σ_1_ = 0.12, σ_3_ = 0.038 nm). A reason for the higher microstructural disorder is the higher content of non-carbon atoms in the resin structure, which hinders the carbonization process and the growth of stacks. Since the resole type of carbon is based on formaldehyde and phenol, a higher oxygen content as well as a higher amount of sp^3^-hybridized carbon atoms, which connect the phenolic groups, is expected. The results show that the pitch-based monolithic exhibits a more ordered microstructure, consisting of larger graphene stacks and similar layer sizes, than the monolithic resin.

## Conclusion

In this study the hard-templating of carbon materials using a meso-macroporous SiO_2_ scaffold is investigated with respect to the impact of two different, commonly used carbon precursors, namely pitch and resin. The samples were heat treated at 800 and 3000 °C, which corresponds to the extreme cases of a typical carbonization temperature (800 °C) and a temperature (3000 °C) that usually leads to graphitization. Thus, the investigation of these four carbon materials allowed for an elucidation of the impact of temperature-induced carbonization and graphitization on two structural features, namely the porosity and the concomitant evolution of the graphene stacks. The final carbon monoliths were analyzed with regard to the relationship between meso- and macroporosity and the sp^2^-hybridized atomic structure, the latter being determined by the carbon source. Based on porosity analysis (Hg porosimetry and physisorption), small-angle neutron scattering, wide-angle X-ray scattering and Raman spectroscopy we find substantial differences in the porosity between the two types of carbon materials on all three levels of porosity, i.e., micro-, meso- and macroporosity. While the average macropore sizes are similar, owing to the identical SiO_2_ scaffold used, the homogeneity of the macropore space of the resin-derived carbon is lower, probably being a consequence of polycondensation reactions rupturing the carbonaceous network.

The more interesting disparity concerns the voids at the nanometer range. For both types of carbon material, physisorption analysis reveals mesopores in the range of 7–12 nm, which corresponds well to the mesopore space of the SiO_2_ hard template. The mesopore size distribution of the resin-based sample is more defined, resembling the one of the SiO_2_ monolith. This indicates a more precise replication than with the pitch precursor. This finding is plausible, as the graphene stacks in the resin precursor are smaller than the graphene stacks in pitches. In addition, both carbon materials contain a certain accessible micropore volume, which however is considerably smaller than the physisorption-based mesopore volume.

The in situ SANS experiments using deuterated *p*-xylene as contrast matching fluid allowed for the quantification of the volume of accessible and inaccessible micro- and mesopores in relation to the carbon precursor and the heat treatment. Interestingly, a thorough analysis of the SANS data using the chord-length distribution (CLD) concept reveals a subtle picture of these nanometer-sized voids. For the pitch-based porous carbon the average pore size is ca. 7 nm, corresponding well to Ar physisorption analysis. Since SANS probes accessible and inaccessible voids, all mesopores are thus accessible. The pitch precursor exhibits a phase transformation during carbonization at temperatures between 350 and 500 °C and forms a liquid-crystal-like state, the lower viscosity of which prevents the formation of closed voids [15]. By contrast, SANS analysis of the empty resin probe (800 °C) provides an average pore size of ca. 1 nm, which is substantially smaller than the average pore size obtained from Ar physisorption. Hence, this the templated resin-based carbon contains a significant concentration of inaccessible voids on the nanometer scale. At 3000 °C the resin-based carbon still contains a considerable content of meso- and micropores, amounting to an accessible micro-mesopore volume of 0.2 mL/g. This quite high porosity is probably due to the fact that resin yields non-graphitizable carbon, i.e., cannot be converted into graphite, even at high temperature. The pores are formed due to the rigid network-like connection of the aromatic groups and the more isotropic arrangement of the resin stacks compared to the anisotropic arrangement of the pitch stacks. Furthermore, the condensation of the resin precursor molecules generates water, which might contribute to the formation of closed voids.

**Figure 8:**
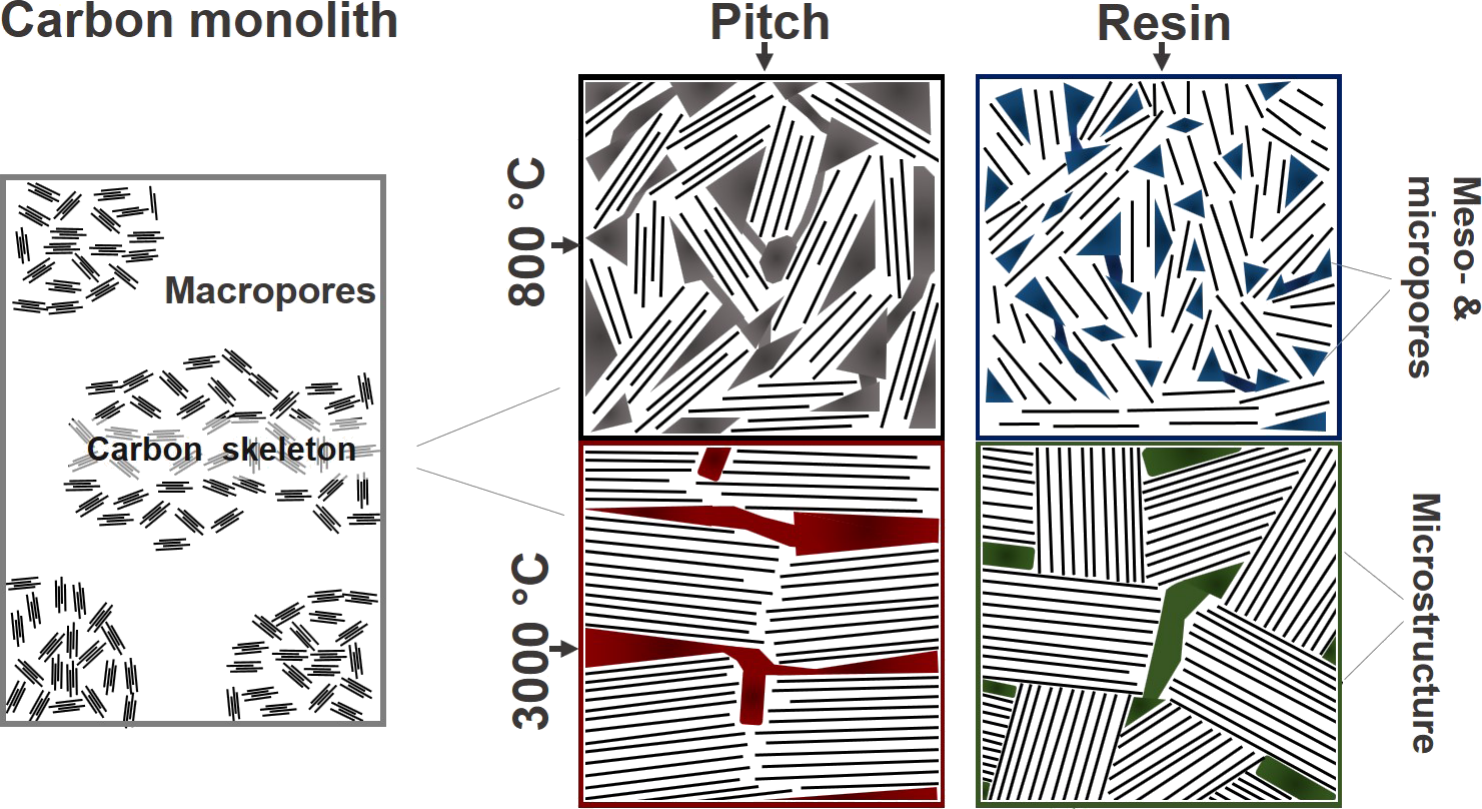
Structural evolution of the different carbon materials in the empty state in comparison to the bulk (non-templated) resin treated at the same temperatures (800 and 3000 °C).

However, surprisingly even the pitch-based carbon does not form graphite, which is counterintuitive as pitches belong to the carbon materials that usually transform readily into graphite. This inhibition of graphite formation is probably due to the confinement effect of the nanostructure imposed by the nanoscale porosity. The structural alterations on the nanometer scale are depicted in Figure 8, emphasizing the relationship between the graphene stacks (size) and the changes in size and accessibility of micro- and mesopores. Still, these materials possess a large macropore volume, proving that the templating procedure provides an almost perfect 1:1 copy of the pristine SiO_2_ macropore space.

Overall, this study provides insight into the evolution of templated nanoscaled porosity in carbon materials in relation to the growth of the graphene stacks and their conversion into graphite. One major finding, which was obtained from the combination of physisorption and in situ SANS is the discrepancy in the accessible micro/mesoporosity between Ar and deuterated *p*-xylene. DPX serves as a representative substance for relevant applications (e.g., supercapacitors and filtering), since its molecule size is more comparable to common organic electrolytes such as tetrahydrofuran [58], acetonitrile [59], ethylene carbonate [60], propylene carbonate [61] and ionic liquids [1] in contrast to argon. Furthermore, the adsorption of xylene isomers is an important step for synthesizing polyethylene terephthalate (PET) and polybutylene terephthalate (PBT) [62]. Moreover, Ar physisorption measurements are conducted at 87 K, which is a less suitable temperature for applications compared to DPX adsorption measurements at 286 K.

The deviation was found for two different carbon precursors (pitch, resin), which can be regarded as representatives in regard to carbon precursors in general. These differences essentially indicate that physisorption using probe gases such as Ar or N_2_ can provide misleading parameters (surface area, volume, pore size) if to be used to appraise the accessibility of the nanoscaled pore space.

## Experimental

The synthesis of the silica monolith is based on the Nakanishi process [37–38]. The silica templates were infiltrated by two different carbon precursors, namely a mesophase pitch and a formaldehyde resole. The template was removed with hydrofluoric acid. Further information can be found in a recent study [41].

SANS data with a very good signal-to-noise ratio were acquired utilizing the V16 instrument on the cold neutron guide hall of BER-II at the Helmholtz-Zentrum Berlin [63]. Using detector distances of 1.7 m and 11.23 m with the respective chopper and collimator settings resulted in a neutron flux of 2 × 10^6^ cm^−2^·s^−1^. The range of the scattering vector *s* was 0.008–0.9 nm^−1^. Background measurements required for proper data reduction were performed (empty cell, water, Cd aperture). All samples were measured in DEGAS copper cells, exhibiting a sample volume of 0.2545 cm^3^, at 286 K in vacuum (empty) or filled with deuterated *p*-xylene (DPX, C_8_D_10_) atmosphere (*p*_0_ = 5.7 mbar). The obtained raw data was corrected by subtracting the sample cell backgrounds and reduced by using the software MANTID. The intensity of the scattering was obtained in absolute units of cm^−1^.

Ar physisorption measurements were conducted at 87 K using an AutoSorb iQ instrument coupled with a CryoSync add-on. The samples were outgassed under vacuum at 120 °C for at least 12 h. The pore-size distributions were calculated by quenched solid-state functional theory (QSDFT) for carbon, using the Quantachrome ASiQwin software and the model “Ar at 87 K on carbon materials (cylindr./sphere pores QSDFT adsorption branch)”.

Mercury intrusion porosimetry (MIP) measurements were conducted using a Pascal 140/440 from Thermo Scientific.

A scanning electron microscope type Merlin from Zeiss was used to obtain pictures of the monolithic morphologies.

The wide-angle X-ray scattering data were measured with a PANalayitcal X’Pert Pro powder diffractometer. A wavelength of λ = 0.15418 nm was used. The 2θ measurement range was from 10° to 100°. A no-background single-crystal sample holder made of silicon was used.

## Supporting Information

File 1 SEM images of the silica template and the carbon monolith (800 °C).

File 2 Formulae used for the calculation of parameter based on the SANS data.

## References

[1] Balducci A, Dugas R, Taberna P L, Simon P, Plée D, Mastragostinoc M, Passerini S (2007). J Power Sources.

[2] Portet C, Taberna P L, Simon P, Flahaut E, Laberty-Robert C (2005). Electrochim Acta.

[3] Ji X, Lee K T, Nazar L F (2009). Nat Mater.

[4] Jayaprakash N, Shen J, Moganty S S, Corona A, Archer L A (2011). Angew Chem, Int Ed.

[5] Radosz M, Hu X, Krutkramelis K, Shen Y (2008). Ind Eng Chem Res.

[6] Ao C H, Lee S C (2005). Chem Eng Sci.

[7] Reungoat J, Escher B I, Macova M, Argaud F X, Gernjak W, Keller J (2012). Water Res.

[8] Hameed B H, Din A T M, Ahmad A L (2007). J Hazard Mater.

[9] Nishihara H, Kyotani T (2012). Adv Mater (Weinheim, Ger).

[10] Xia Y, Yang Z, Mokaya R (2010). Nanoscale.

[11] Inagaki M, Orikasa H, Morishita T (2011). RSC Adv.

[12] Inagaki M, Toyoda M, Soneda Y, Tsujimura S, Morishita T (2016). Carbon.

[13] Inagaki M, Toyoda M, Tsumura T (2014). RSC Adv.

[14] Oberlin A (1984). Carbon.

[15] Loeh M O, Badaczewski F, Faber K, Hintner S, Bertino M F, Mueller P, Metz J, Smarsly B M (2016). Carbon.

[16] Bonnamy S (1999). Carbon.

[17] Ouzilleau P, Gheribi A E, Chartrand P (2016). Carbon.

[18] Gadiou R, Didion A, Saadallah S-E, Couzi M, Rouzaud J-N, Delhaes P, Vix-Guterl C (2006). Carbon.

[19] Marie J, Mering J, Walker P L (1970). Graphitization of soft carbon materials. Chemistry and Physics of Carbon.

[20] Auguie D, Oberlin M, Oberlin A, Hyvernat P (1980). Carbon.

[21] Warren B E (1941). Phys Rev.

[22] Fitzer E, Kochling K-H, Boehm H P, Marsh H (1995). Pure Appl Chem.

[23] Franklin R E (1951). Proc R Soc London, Ser A.

[24] Bourrat X, Oberlin A, Escalier J C (1986). Fuel.

[25] Shi H, Reimers J N, Dahn J R (1993). J Appl Crystallogr.

[26] Azuma H (1998). J Appl Crystallogr.

[27] Fujimoto H, Shiraishi M (2001). Carbon.

[28] Ruland W, Smarsly B (2002). J Appl Crystallogr.

[29] Puech P, Dabrowska A, Ratel-Ramond N, Vignoles G L, Monthioux M (2019). Carbon.

[30] Faber K, Badaczewski F, Oschatz M, Mondin G, Nickel W, Kaskel S, Smarsly B M (2014). J Phys Chem C.

[31] Faber K, Badaczewski F, Ruland W, Smarsly B M (2014). Z Anorg Allg Chem.

[32] Pfaff T, Badaczewski F M, Loeh M O, Franz A, Hoffmann J-U, Reehuis M, Zeier W G, Smarsly B M (2019). J Phys Chem C.

[33] Ahmadpour A, Do D D (1996). Carbon.

[34] Lillo-Ródenas M A, Cazorla-Amorós D, Linares-Solano A (2003). Carbon.

[35] Nakanishi K, Tanaka N (2007). Acc Chem Res.

[36] Nakanishi K (1997). J Porous Mater.

[37] Adelhelm P, Cabrera K, Smarsly B M (2012). Sci Technol Adv Mater.

[38] Loeh M O, Badaczewski F, von der Lehr M, Ellinghaus R, Dobrotka S, Metz J, Smarsly B M (2018). Carbon.

[39] Doherty C M, Caruso R A, Smarsly B M, Adelhelm P, Drummond C J (2009). Chem Mater.

[40] Kim Y-S, Guo X-F, Kim G-J (2010). Catal Today.

[41] Yu L, Brun N, Sakaushi K, Eckert J, Titirici M M (2013). Carbon.

[42] Reitz C, Breitung B, Schneider A, Wang D, von der Lehr M, Leichtweiss T, Janek J, Hahn H, Brezesinski T (2016). ACS Appl Mater Interfaces.

[43] Schneider A, Weidmann C, Suchomski C, Sommer H, Janek J, Brezesinski T (2015). Chem Mater.

[44] Mascotto S, Kuzmicz D, Wallacher D, Siebenbürger M, Clemens D, Risse S, Yuan J, Antonietti M, Ballauff M (2015). Carbon.

[45] Perret R, Ruland W (1972). J Appl Crystallogr.

[46] Perret R, Ruland W (1968). J Appl Crystallogr.

[47] Ruland W (2010). J Appl Crystallogr.

[48] Smarsly B, Antonietti M, Wolff T (2002). J Chem Phys.

[49] Harris P J F (1997). Int Mater Rev.

[50] Tzeng S-S, Chr Y-G (2002). Mater Chem Phys.

[51] Sharma S, Shyam Kumar C N, Korvink J G, Kübel C (2018). Sci Rep.

[52] Jurkiewicz K, Pawlyta M, Zygadło D, Chrobak D, Duber S, Wrzalik R, Ratuszna A, Burian A (2018). J Mater Sci.

[53] Jenkins G M, Kawamura K (1971). Nature.

[54] Jenkins G M, Kawamura K, Ban L L (1972). Proc R Soc London, Ser A.

[55] Fitzer E, Schäfer W (1970). Carbon.

[56] Badaczewski F, Loeh M O, Pfaff T, Dobrotka S, Wallacher D, Clemens D, Metz J, Smarsly B M (2019). Carbon.

[57] Thommes M, Kaneko K, Neimark A V, Olivier J P, Rodriguez-Reinoso F, Rouquerol J, Sing K S W (2015). Pure Appl Chem.

[58] Alcántara R, Lavela P, Ortiz G F, Tirado J L (2005). Electrochem Solid-State Lett.

[59] Lust E, Jänes A, Pärn T, Nigu P (2004). J Solid State Electrochem.

[60] Laheäär A, Kurig H, Jänes A, Lust E (2009). Electrochim Acta.

[61] Hahn M, Würsig A, Gallay R, Novák P, Kötz R (2005). Electrochem Commun.

[62] Yang Y, Bai P, Guo X (2017). Ind Eng Chem Res.

[63] Vogtt K, Siebenbürger M, Clemens D, Rabe C, Lindner P, Russina M, Fromme M, Mezei F, Ballauff M (2014). J Appl Crystallogr.

