# Correction: An advanced structural characterization of templated meso-macroporous carbon monoliths by small- and wide-angle scattering techniques

**DOI:** 10.3762/bjnano.11.54

**Published:** 2020-04-22

**Authors:** Felix M Badaczewski, Marc O Loeh, Torben Pfaff, Dirk Wallacher, Daniel Clemens, Bernd M Smarsly

**Affiliations:** 1 Institute of Physical Chemistry, Justus Liebig University, Heinrich-Buff-Rung 17, 35492 Giessen, Germany; 2 Schunk Carbon Technology GmbH, Rodheimer Straße 59, 35452 Heuchelheim, Germany; 3 Helmholtz-Zentrum Berlin für Materialien und Energie, Hahn-Meitner-Platz 1, 14109 Berlin, Germany,; 4 Center for Materials Research (LaMa), Justus-Liebig-University, Heinrich-Buff-Ring 16, 35392 Giessen, Germany

**Keywords:** adsorption, carbon materials, mesoporosity, microporosity, microstructure, pore structure, small-angle neutron scattering (SANS), wide-angle X-ray scattering (WAXS)

The following graph (A) should be implemented in Figure 4 of the original article, since it was part of the manuscript and was accidently removed during the revision process. No other change in the corresponding text and caption of Figure 4 is necessary.

**Figure 1:**
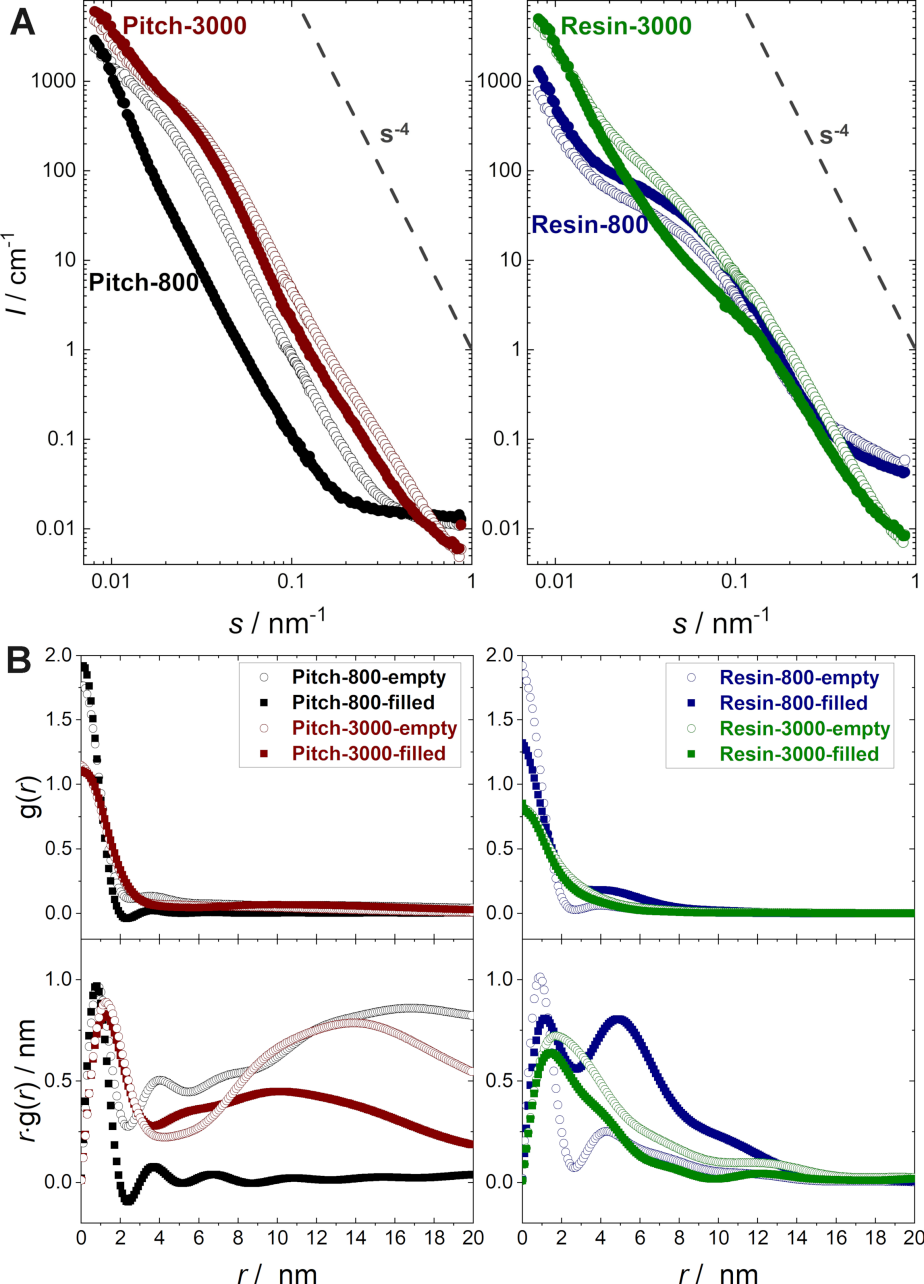
**Figure 4 in the original article:** SANS raw data (A) and CLD analysis (B) for the four resin- and pitch-based carbon materials, treated at 800 °C and 3000 °C. For all samples, SANS analysis was performed on evacuated samples (hollow symbols), as well as under a maximum load of deuterated *p*-xylene (filled symbols). All samples exhibit a Porod-asymptote (*s^−4^*) at large *s* (modulus of the scattering vector), proving an almost ideal two-phase system (pore–carbon) with sharp interfacial boundaries on the nanometer scale.

