# Supplementary material for: An advanced structural characterization of templated meso-macroporous carbon monoliths by small- and wide-angle scattering techniques
Source: Beilstein J Nanotechnol. 2020 Feb 10;11:310–22. doi: 10.3762/bjnano.11.23 (PMC7034224; doi:10.3762/bjnano.11.23)
Supplement: File 1 — SEM images of the silica template and the carbon monolith (800 °C). [file Beilstein_J_Nanotechnol-11-310-s001.pdf]

BEILSTEIN JOURNAL OF NANOTECHNOLOGY

Supporting Information

for

An advanced structural characterization of templated meso-macroporous carbon monoliths by small- and wide-angle scattering techniques

Felix M. Badaczewski, Marc O. Loeh, Torben Pfaff, Dirk Wallacher, Daniel Clemens and Bernd M. Smarsly

Beilstein J. Nanotechnol. **2020,** *11,* 310–322. doi:10.3762/bjnano.11.23

SEM images of the silica template and the carbon monolith (800 °C)

License and Terms: This is a supporting information file under the terms of the Creative Commons Attribution License (https://creativecommons.org/licenses/by/4.0). Please note that the reuse, redistribution and reproduction in particular requires that the authors and source are credited.
The license is subject to the *Beilstein Journal of Nanotechnology* terms and conditions: (https://www.beilstein-journals.org/bjnano)

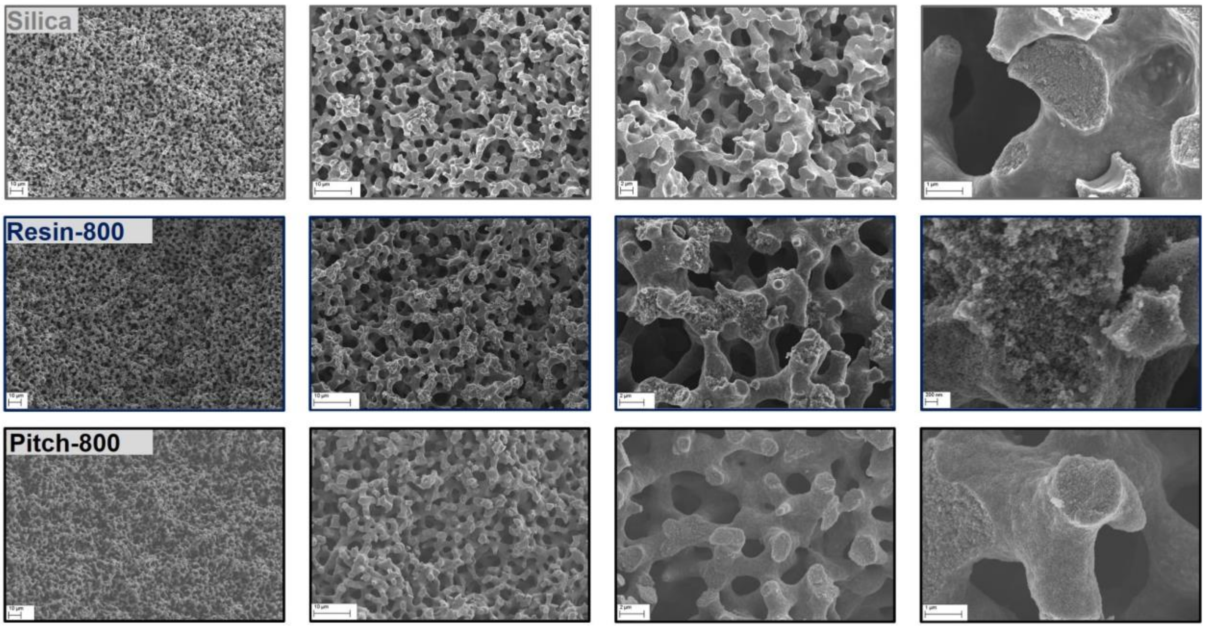

Figure S1: SEM pictures of the silica template (grey), the resin-based (blue) and the pitch-based (black) monolith carbonized at 800 °C.
