# Supplementary material for: An advanced structural characterization of templated meso-macroporous carbon monoliths by small- and wide-angle scattering techniques
Source: Beilstein J Nanotechnol. 2020 Feb 10;11:310–22. doi: 10.3762/bjnano.11.23 (PMC7034224; doi:10.3762/bjnano.11.23)
Supplement: File 2 — Formulae used for the calculation of parameter based on the SANS data. [file Beilstein_J_Nanotechnol-11-310-s002.pdf]

BEILSTEIN JOURNAL OF NANOTECHNOLOGY

# Supporting Information

for

# An advanced structural characterization of templated meso-macroporous carbon monoliths by small- and wide-angle scattering techniques

Felix M. Badaczewski, Marc O. Loeh, Torben Pfaff, Dirk Wallacher, Daniel Clemens and Bernd M. Smarsly

*Beilstein J. Nanotechnol.* **2020,** *11,* 310–322. doi:10.3762/bjnano.11.23

# Formulae used for the calculation of parameter based on the SANS data

License and Terms: This is a supporting information file under the terms of the Creative Commons Attribution License (https://creativecommons.org/licenses/by/4.0). Please note that the reuse, redistribution and reproduction in particular requires that the authors and source are credited.
The license is subject to the *Beilstein Journal of Nanotechnology* terms and conditions: (https://www.beilstein-journals.org/bjnano)

Formulae used for the calculation of parameter describing the carbon pore structure based on the SANS data [1,2]:

$$k = 4\pi \int_0^\infty s^2\, I(s)\, \mathrm{d}s = \mathrm{V}\varrho_{carbon}^2 (1-P)P \quad \text{(S1)}$$

$k$: normalized Porod invariant

$s$: scattering vector

$I(s)$: scattering intensity

$V$: irradiated volume

$\varrho_{carbon}$: neutron scattering length density of carbon

$P$: porosity

$$g(r) = -8\int_0^\infty \left(1 - \frac{2\pi^3 s^4 l_p I(s)}{k}\right) \frac{d^2}{d(2\pi r s)^2}\left(\frac{\sin(2\pi r s)}{2\pi r s}\right) ds = \sum_{j=0}^{N} a_j g_j(\frac{r}{b}) \quad \text{(S2)}$$

$$g(r) = \sum_{j=0}^{N} a_j g_j(\frac{r}{b}) \quad \text{(S3)}$$

$$g_j(r) = \frac{1}{j!}\left(\frac{r}{b}\right)^j e^{\left(-\frac{r}{b}\right)^m} \quad \text{(S4)}$$

$g(r)$: chord length distribution (CLD)

$l_p$: Porod length

$a_j$, $g_j$: fit parameters

$$l_\mathrm{p} = \int_0^\infty \mathrm{r}\, \mathrm{g}(r)\, \mathrm{d}r \quad \text{(S5)}$$

$$l_\mathrm{p} = \frac{2\int_0^\infty s^2\, I(s)\, \mathrm{d}s}{\pi^2 \lim\limits_{s\to\infty} s^4\, I(s)} \quad \text{(S6)}$$

$$l_{\text{pores}} = \frac{l_{\text{p}}}{1-P} \qquad \text{(S7)}$$

$l_{\text{pores}}$: average pore size

$$l_{\text{matter}} = \frac{l_{\text{p}}}{P} \qquad \text{(S8)}$$

$l_{\text{matter}}$: average distance between pore walls

$$\frac{S}{V} = \frac{4\,P\,(1-P)}{l_{\text{p}}} \qquad \text{(S9)}$$

$\frac{S}{V}$: interface

$$\kappa_{\text{l}} = \frac{l_{\text{c}}}{l_{\text{p}}} - 1 \qquad \text{(S10)}$$

$\kappa_{\text{l}}$: polydispersity

## References


1. Smarsly, B.; Antonietti, M.; Wolff, T. *J. Chem. Phys.* **2002,** *116,* 2618–2627. doi:10.1063/1.1433463

2. Badaczewski, F.; Loeh, M. O.; Pfaff, T.; Dobrotka, S.; Wallacher, D.; Clemens, D.; Metz, J.; Smarsly, B. M. *Carbon* **2019,** *141,* 169–181. doi:10.1016/j.carbon.2018.09.025
